# Signature MicroRNA expression profile is associated with lipid metabolism in African green monkey

**DOI:** 10.1186/s12944-019-0999-2

**Published:** 2019-02-28

**Authors:** Xiao-jun Zhou, Jin Wang, Hua-hu Ye, Yun-zhi Fa

**Affiliations:** Laboratory Animal Center, the Academy of Military Medical Sciences, Beijing, 100071 People’s Republic of China

**Keywords:** Lipid metabolism, microRNAs, African green monkey, Cynomolgous macaque

## Abstract

**Background:**

Non-human primates (NHPs) are important models of medical research on obesity and cardiovascular diseases. As two of the most commonly used NHPs, cynomolgus macaque (CM) and African green monkey (AGM) own different capacities in lipid metabolism of which the mechanism is unknown. This study investigated the expression profiles of lipid metabolism-related microRNAs (miRNAs) in CM and AGM and their possible roles in controlling lipid metabolism-related gene expression.

**Methods:**

By small RNA deep sequencing, the plasma miRNA expression patterns of CM and AGM were compared. The lipid metabolism-related miRNAs were validated through quantitative reverse-transcription (RT) polymerase chain reaction (PCR). Related**-**target genes were predicted by TargetScan and validated in Vero cells.

**Results:**

Compared to CM, 85 miRNAs were upregulated with over 1.5-fold change in AGM of which 12 miRNAs were related to lipid metabolism. miR-122, miR-9, miR-185, miR-182 exhibited the greatest fold changes(fold changes are 51.2, 3.8, 3.7, 3.3 respectively; all *P* < 0.01). And 77 miRNAs were downregulated with over 1.5-fold change in AGM of which 3, miR-370, miR-26, miR-128 (fold changes are 9.3, 1.8, 1.7 respectively; all *P* < 0.05) were related to lipid metabolism. The lipid metabolism-related gene targets were predicted by TargetScan and confirmed in the Vero cells.

**Conclusion:**

We report for the first time a circulating lipid metabolism-related miRNA profile for CM and AGM, which may add to knowledge of differences between these two non-human primate species and miRNAs’ roles in lipid metabolism.

**Electronic supplementary material:**

The online version of this article (10.1186/s12944-019-0999-2) contains supplementary material, which is available to authorized users.

## Background

As short (≈22-26 nt) endogeous RNA molecules that regulate gene expression at the post-transcriptional levels by binding to the 3′ untranslated regions (UTRs) of their target mRNAs [1], miRNAs have been described as vital regulators of several biological processes including developmental and metabolic functions. In many pathological conditions such as obesity, type 2 diabetes, cardiovascular diseases, hypertension and cancers, the concentration of miRNA in circulation has been found to be altered [2], suggesting regulatory role in maintaining metabolism homeostasis in humans [3–5].

Non-human primates (NHPs) have been vital for medical research due to their close evolutionary relationship, similar behavioral and physiological characteristics to humans. Two of the most commonly used NHPs are Cynomolgus macaque (*Macaca fascicularis*) and African green monkey (*Chlorocebus Sabaeus*), which can both be used in studies of neuroscience, infectious diseases and drug safety testing [6–10]. Differences also exist between these two species. For example, AGM has been known to be resistant to simian immunodeficiency virus which can be used as a model organism for HIV research [11, 12]. Also, AGM develops spontaneous hypertension with pathophysiological changes that mimic those of patients with essential hypertension [13, 14]. Research has also shown that genetic factors influence the atherogenic response of lipoproteins to dietary fat and cholesterol in nonhuman primates which reflects different capacities in lipid metabolism between AGM and CM [15].

This study aims to identify the differently expressed miRNAs and their potential targets related to lipid metabolism between AGM and CM. Understanding the roles of miRNAs in lipoprotein and lipid metabolism may eventually lead to development of new disease biomarkers or therapeutic strategies.

## Methods

### Animal care and housing

All protocols were in strict accordance with the recommendations in the Guide for the Care and Use of Laboratory Animals. Animals were housed in troop enclosures with an outdoor facility amd fed nonhuman primate chow per day and a combination of fresh bananas, apples, carrots 3 days per week. As shown in Additional file 1: Table S1, 16 animals were used of which 6 were used for miRNA profiling with the others used miRNA validation.

### Plasma collection and lipid level analysis

All animals were fasted overnight and anesthetized with intramuscular injection (10 to 15 mg/kg). Blood samples (5 ml) of CM and AGM were collected from the femoral vein and drawn into EDTA-containing tubes held on ice. Part of the blood samples were separated into plasma and cellular fractions by centrifugation at 1000 g for 10 min. And the plasma lipid levels were analyzed by an automated chemistry analyzer (Olympus AU5800, Beckman Coulter).

### RNA isolation and small RNA deep sequencing

Total plasma RNA was harvested from the rest blood samples with the TRI Reagent BD (Sigma Aldrich) and the RNeasy mini kit (Qiagen) according to the manufacturers’ instructions. In detail, 250 μL EDTA-containing plasma was transferred to an Eppendorf tube and mixed thoroughly with TRI reagent, incubated for 5 min at room temperature, and subsequently mixed with 140 μL chloroform. The aqueous phase containing the RNA was carefully removed, and RNA was precipitated by addition of 100% ethanol. The mixture was applied to an RNeasy Mini spin column and washed several times, and RNA was eluted by addition of 25 μL RNase-free water. RNA was stored at − 80 °C until further processing. Total RNA (4 to 8 μg) was size fractionated and the small RNAs (< 300 nucleotides) were isolated and 3′ extended with a poly(A) tail using poly(A) polymerase. An oligonucleotide tag was then ligated to the poly(A) tail for later fluorescent-dye staining. Hybridization was performed overnight on a microfluidic chip provided by Shanghai KangChen biotechnology company. The hybridization buffer was 6 × SSPE (0.9 M NaCl, 60 mM Na_2_HPO_4_, 6 mM EDTA, pH 6.8) containing 25% formamide at 34 °C. After RNA hybridization, tag-conjugating Cy3 and Cy5 dyes were circulated through the microfludic chip for dye staining. Scanning was performed with the Axon GenePix 4000B microarray scanner. GenePix pro version 6.0 was used to read image raw intensity. The intensity of the the green signal was calculated after background subtraction. The median normalization method was used to acquire normalized data. The threshold value for significance used to define upregulation or downregulation of miRNAs was a fold change > 1.5, with a value of *P* < 0.05 calculated by the *t* test.

### Inhibition of endogenous miRNAs

Locked nucleic acid (LNA)-modified anti-miRs (Exiqon) were used for the inhibition of endogenous miRNAs in African green monkey-derived Vero cells [16]. Vero cells were maintained in DMEM supplemented with 100 U/ml penicillin, 100 μg/ml streptomycin, nonessential amino acids, and 10% fetal bovine serum (FBS) (Invitrogen). Cells were subcultured at 80% confluency for propagation. Vero cells were subcultured onto 96 well plates and transfected at > 90% confluency with LNA-modified anti-miRs which were transfected at a final concentration of 10 nM by Lipofectamine 3000 (Invitrogen).

### Stem-loop miRNA quantitative RT-PCR

First, each mature miRNA was extended and reverse transcribed by a sequence specific stem-loop primer using MMLV reverse transcriptase (Takara). Then the reverse transcribed miRNA was quantified by a fluorescently labeled hybridization probe using the strand replacement reaction. and quantitative PCR was performed by an ABI PRISM7500 system (Applied Biosystems). The PCR conditions were as follows: 95 °C 3 min, 40 cycles of 95 °C1 2 s, 62 °C4 0 s. miRNAs and related target genes implicated in the regulation of lipid metabolism were obtained through the literature [2]. The RT primers and the primer sets specific for each miRNA amplification designed according the mature miRNA sequence obtained from the miRbase database and shown in Additional file 2: Table S2. Selected miRNAs were further quantified with TaqMan quantitative RT-PCR. mRNA expression levels of the lipid metabolism-related genes predicted to be targeted by miRNAs inhibited by anti-miR transfection were measured by qPCR using SYBR green chemistry [17]. Primer sequences for the target genes are listed in Additional file 3: Table S3.

### Western blot analysis

Anti-miR-treated vero cells were lysed in protein sample buffer containing 50 mM Tris-HCl, 150 mM NaCl. 5 mM EDTA, 0.2 mM sodium orthovanadate, 1% Triton X-100, 1% sodium deoxycholate, and 1% sodium dodecyl sulfate; the buffer was also supplemented with aprotinin (2 μg/ml), pepstatin A (0.7 μg/ml), leupeptin (0.5 μg/ml), and PMSF (1 mM). For each sample, 30 μg total protein was electrophoresed through a 10% sodium dodecyl sulfate-polyacrylamide gel and transfered onto a nitrocellulose membrane.The membrane was blocked by pre-incubation with 5% skim milk, incubated with the specific rabbit anti-CPT1A (ThermoFisher) and mouse anti-GAPDH (ThermoFisher) overnight at 4 °C, which were detected with horseradish peroxidase-conjugated secondary antibody (Pierce) and visualized with a chemiluminescent substrate (Pierce).

### Statistical analysis

Quantile normalized values for miRNA in each sample were plotted as fold change in comparison with the mean expression of each miRNA within the control group. Significance was assessed by the t test. Statistical analyses were performed using GraphPad Prism version 5.01.

## Results

### Expression profiles of plasma miRNAs in CM and AGM

**Fig. 1:**
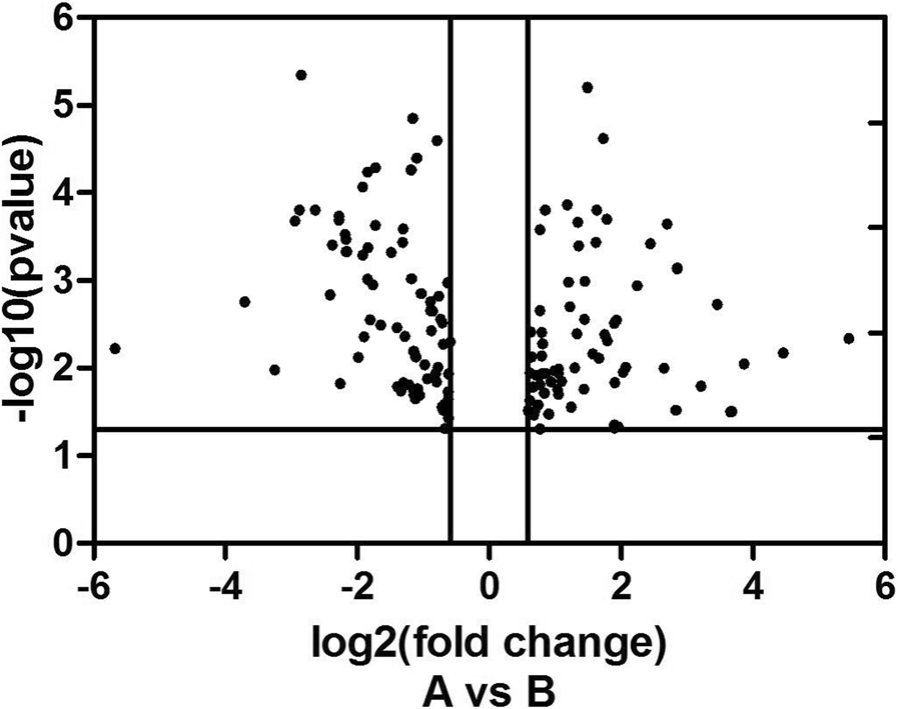
Volcano plot for the differential expressed miRNA in PBMCs of CM and AGM. A stands for miRNAs upregulated with over 1.5 fold change in CM while B stands for miRNAs upregulated with over 1.5 fold change in AGM (*P* < 0.05)

**Fig. 2:**
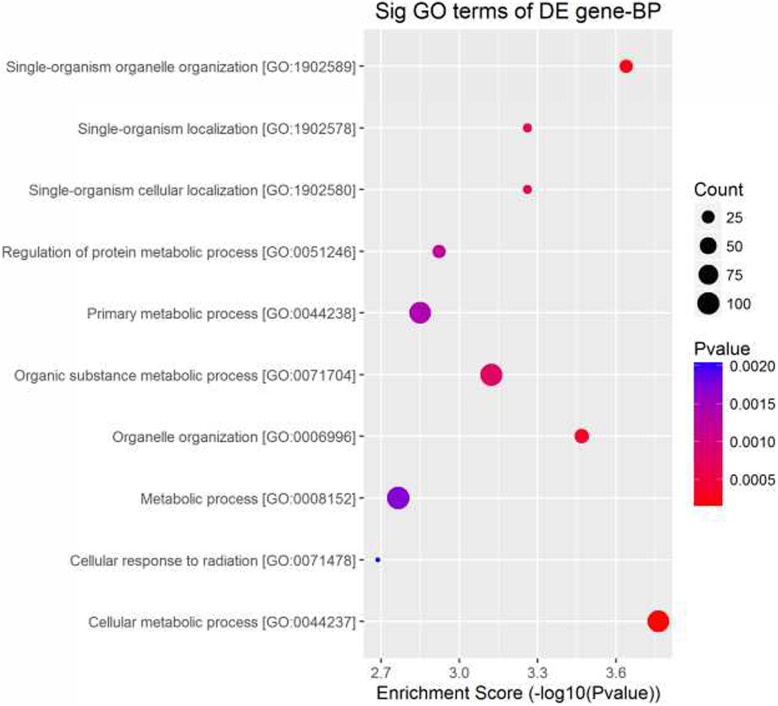
Enrichment map of GO categories for biological process. Colors represent *P* values on a log scale (with red corresponding to the most highly significant, *P* < 0.05). Node size represents the number of genes in a category

As shown in Additional file 1: Table S1, 16 animals were used of which 6 were used for miRNA profiling with the others used miRNA validation. There were no significant differences for weights and plasma lipid levels between the CM and AGM group. We then profiled the plasma miRNA expression in 3 CM and 3 AGM. As shown in Fig. 1, 162 miRNAs exhibited different expression with over 1.5-fold change between CM and AGM. GO analysis for biological process (BP) demonstrated that more genes reside in the categories of metabolic processes (Fig. 2). Among the 85 upregulated miRNAs in AGM, 12 were related to lipid metabolism, while 3 out of 77 downregulated miRNAs were related to lipid metabolism. miR-122 and miR-370 own the greatest upregulated and donwregulated fold changes (*P* < 0.05), respectively. miRNAs with over 2-fold change related to lipid metabolism are shown in Table 1.

**Table 1:** Properties of lipid metabolism-related miRNAs expressed with over 2 fold change in PBMCs of CM and AGM through small RNA sequencing

Upregulated miRNAs	Fold change (AGM/CM)	*P*	Downregulated miRNAs	Fold change (CM/AGM)	*P*
hsa-miR-122-5p	51.2	0.006	hsa-miR-370-3p	9.3	0.016
hsa-miR-9-5p	3.8	0.0005			
hsa-miR-185-5p	3.7	0.004			
hsa-miR-182-5p	3.3	0.0002			
hsa-miR-181a-5p	2.6	0.003			
hsa-miR-144-3p	2.3	0.016			
hsa-miR-10b-5p	2.2	0.022			
hsa-miR-148a-3p	2.2	0.02			
hsa-miR-130b-5p	2.0	0.001			

### Validation of miRNA expression in CM and AGM

**Fig. 3:**
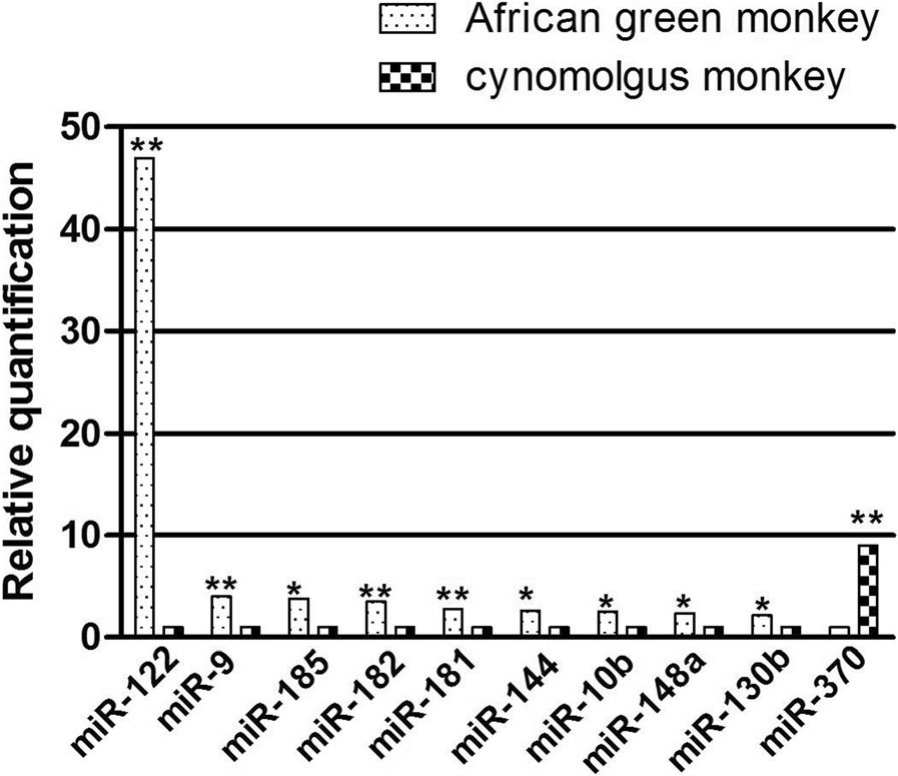
Independent validation of differential expression of miRNA in PBMCs of CM and AGM. Quantitative reverse-transcription polymerase chain reaction for miRNAs in an independent validation set of 5 CMs and 5 AGMs. The relative expression of each miRNA in AGM compared with CM was normalized to U6 expression. The *P* values were calculated by 2-sided Student *t* test. * stands for *P* < 0.05 while ** stands for *P* < 0.01

To validate the profiling data, miRNAs in Table 1 were further analyzed via quantitative RT-PCR between plasma samples from 5 CM and 5 AGM. Results showed a 48.0-fold increase for hsa-miR-122-5p (*P* < 0.01), a 3.0~4.0-fold increase for hsa-miR-9-5p, hsa-miR-185-5p, hsa-miR-182-5p (all *P* < 0.05), a 2.0~3.0-fold increase for hsa-miR-181-5p, hsa-miR-144-3p, hsa-miR-10b-5p, hsa-miR-148a-3p, hsa-miR-130b-5p (all *P* < 0.05), and an ≈9.5 decrease for hsa-miR-370-3p (*P* < 0.01) (Fig. 3).

### Effects of selected miRNAs on the lipid metabolism-related target genes

**Fig. 4:**
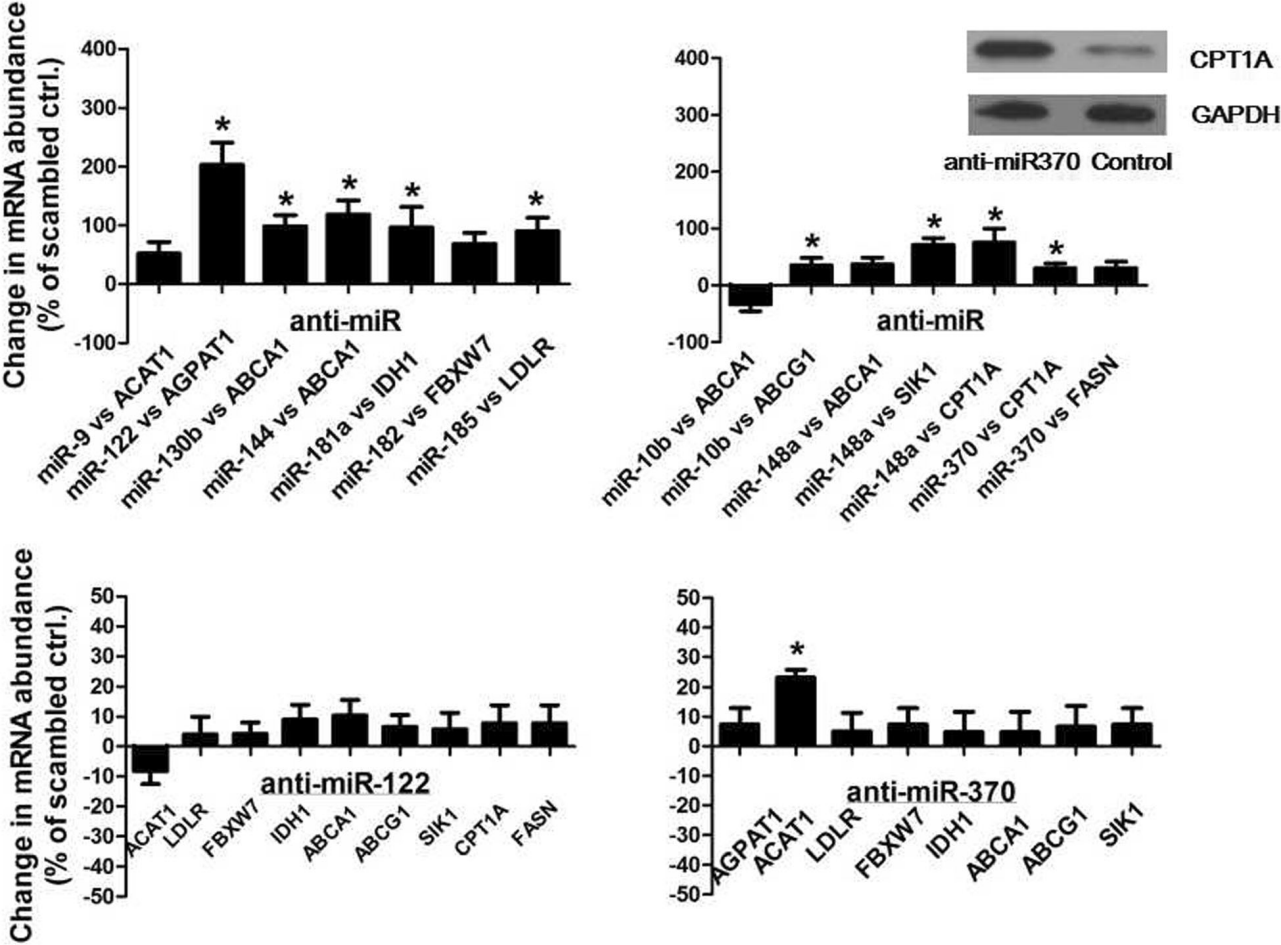
Effect of anti-miR transfections in vero cells on the abundance of mRNAs encoded by lipid metabolism-related genes. Vero cells were transfected with the indicated LNA-modified anti-miRs or scambled anti-miRs (10 nmol/L) for 48 h. mRNA abundance for predicted targets genes was measured by real-time reverse transcription polymerase chain reaction (PCR) and expressed as % change relative to cells treated with scrambled anti-miR on the same quantitative PCR plate.*n* = 3–6.*P < 0.05 vs scrambled anti-miR

**Fig. 5:**
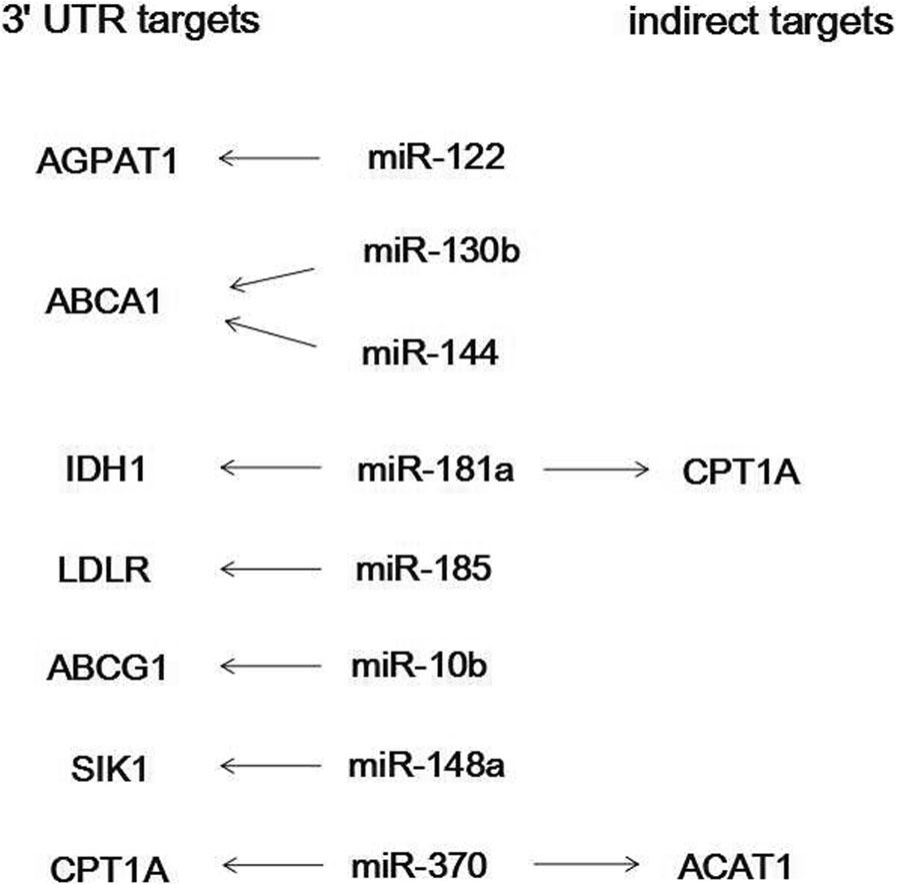
Schematic show of putative mechanisms through which the up- and down-regulation of specific miRNA can affect lipoprotein metabolism

The potential lipid metabolism-related genes targeted by the above miRNAs were selected and analyzed (Table 2). The potential miRNA-target pairs were examined by inhibiting these endogenous miRNAs in Vero cells individually using LNA-modified anti-miRs. Anti-miR treatment may cause the increase on the abundance of mRNA encoded by lipid metabolism-related genes if the predicted target mRNA is indeed suppressed by the endogenous miRNA via mRNA degradation. As shown in Fig. 4, the validated target of hsa-miR-122-5p, hsa-miR-185-5p, hsa-miR-181a-5p, hsa-miR-10b-5p, hsa-miR-370-3p was AGPAT1, LDLR, IDH1, ABCG1, CPT1A, respectively. ABCA1 was the target of both hsa-miR-144-3p and hsa-miR-130b-5p. The validated targets of hsa-miR-148a-3p included SIK1 and CPT1A. The ABCA1 target mRNA was downregulated after anti-miR treatment, suggesting unconventional effects of hsa-miR-10b-5p. Also, we tested whether the effects of anti-miR treatment on the target genes were similar both at mRNA level and at the protein level. As shown in Fig. 4, anti-miR-370 treatment caused upregulation of CPT1A at the protein level. Further, we tested the effects of every anti-miR treatment on unpredicted targets. As shown in Fig. 4 and Fig. 5, anti-miR-370 treatment caused upregulation of ACAT1 while anti-miR-181a treatment caused upregulation of CPT1A, indicating the indirect targets of miRNA may exist.

**Table 2:** Differentially-expressed miRNAs and their predicted targets relevant to lipid metabolism

miRNA	lipid metabolism-related targets	Number of binding sites in AGM	Number of binding sites in CM
hsa-miR-122-5p	AGPAT1	2	3
hsa-miR-9-5p	ACAT1	1	1
hsa-miR-185-5p	LDLR	1	1
hsa-miR-182-5p	FBXW7	2	2
hsa-miR-181a-5p	IDH1	2	2
hsa-miR-144-3p	ABCA1	6	4
hsa-miR-10b-5p	ABCA1	2	1
	ABCG1	1	1
hsa-miR-148a-3p	ABCA1	1	2
	LDLR	0	2
	SIK1	2	2
	CPT1A	2	1
hsa-miR-130b-5p	ABCA1	6	6
	LDLR	0	1
hsa-miR-370-3p	CPT1A	2	1
	FASN	10	12

## Discussion

As regulators of several physiological processes including developmental and metabolic functions, miRNAs play central role in regulating lipoprotein metabolism and cholesterol homeostasis [18–21]. Many pathological conditions such as familial hypercholesterolemia, cardiovascular diseases, obesity, type 2 diabetes occur when the circulation miRNA concentration alters. miRNAs are stable in plasma and may be suitable as biomarkers in cancers and other diseases [22, 23].

NHPs shares similar characteristics in many aspects to humans when they are used in models of neuroscience, infectious diseases and drug safety testing. And differences also exist between these NHP species like CM and AGM. AGM are resistant to simian immunodeficiency virus and can be used as a model spontaneous hypertension. Besides, genetic differences between CM and AGM influence the diet responsiveness [15]. Although draft genome sequences and transcriptome reconstruction and annotation for CM and AGM have been published [24–26], comparison of miRNA profiling data between these two species lacks.

In this study, we compared the circulating miRNA profile between CM and AGM. Several miRNAs related to lipid metabolism exhibited expression with different levels. miR-122 was significantly upregulated with the greatest fold change in AGM. As the most highly expressed miRNA in the liver, miR-122 has been found to regulate liver lipid metabolism [27–29]. Knockdown of miR-122 in mice decreased expression of genes important for cholesterol biosynthesis, whereas adenoviral overexpression of miR-122 increased cholesterol biosynthesis [30]. miR-122 was found to be downregulated in patients with nonalcoholic fatty liver disease (NAFLD) and knockdown of miR-122 in HepG2 cells recapitulated the lipogenic gene expression profile observes in patients with NAFLD [31]. Thus, we conclude that upregulation of miR-122 renders AGM more capable, compared to CM, in regulating lipoprotein metabolism and cholesterol homeostasis. The validated target of miR-122 in vero cells was AGPAT1 which may cause effect on TG biosynthesis and lipid storage. miR-370 has been shown be significantly downregulated in AGM and target CPT1A which facilitates the transfer of long-chain fatty acids across the mitochondrial membrane for β-oxidation. Previous research also demonstrate that miR-370 functions in cholesterol biosynthesis by increasing the level of miR-122 [32]. Whether miR-370 exerts an effect on upregulation of miR-122 in AGM still needs investigation.

Other upregulated miRNAs in AGM include miR-185, miR-181a, miR-10b, miR-144, miR-130b,miR-148a with the validated targets of LDLR, IDH1, ABCG1, ABCA1, SIK1 and CPT1A which function in LDL clearance, regulation of lipid biosynthesis and β-oxidation, HDL biogenesis and cholesterol efflux.

## Conclusion

The present study demonstrates that AGM possess a significantly different miRNA expression profile from CM. The differently expressed miRNAs and their potential targets related to lipid metabolism were investigated, which provide clues that may eventually lead to development of new disease biomarkers or therapeutic strategies.

## Additional files

Additional file 1: **Table S1.** Sex, age, body weight, and plasma lipid information about CMs and AGMs used. 1~3 and 4~6 are CMs and AGMs used for miRNA profiling, respectively. 7~11 and 12~16 are CMs and AGMs used for miRNA validation, respectively. (DOC 44 kb)

Additional file 2: **Table S2.** Sequences of the primers used in the SYBR-green-based quantitative RT-PCR validation. (DOC 48 kb)

Additional file 3: **Table S3.** Primer sequences for miRNA-targeted genes. (DOC 40 kb)

## Abbreviations

ABCA1: ATP-binding cassette subfamily A member 1
ABCG1: ATP-binding cassette subfamily G member 1
ACAT1: acetyl-CoA acetyltransferase 1
AGPAT1: 1-acylglycerol-3-phosphate O-acyltransferase 1
CPT1A: carnitine O-palmitoyltransferase 1
FASN: fatty acid synthase
FBXW7: F-box and WD-40 domain protein 7
IDH1: isocitrate dehydrogenase 1
LDLR: low-density lipoprotein receptor
SIK1: salt-inducible kinase 1
