# Supplementary material for: Signature MicroRNA expression profile is associated with lipid metabolism in African green monkey
Source: Lipids Health Dis. 2019 Feb 28;18:55. doi: 10.1186/s12944-019-0999-2 (PMC6396449; doi:10.1186/s12944-019-0999-2)
Supplement: Supplementary file 1 — Table S1. Sex, age, body weight, and plasma lipid information about CMs and AGMs used. 1~3 and 4~6 are CMs and AGMs used for miRNA profiling, respectively. 7~11 and 12~16 are CMs and AGMs used for miRNA validation, respectively. (DOC 44 kb) [file 12944_2019_999_MOESM1_ESM.doc]

**Additional file 1: Table S1. Sex, age, body weight, and plasma lipid information about CMs and AGMs used. 1~3 and 4~6 are CMs and AGMs used for miRNA profiling, respectively. 7~11 and 12~16 are CMs and AGMs used for miRNA validation, respectively.**

| Animal ID | Sex | Age  (yr) | Body weight  (kg) | TRIG  (mg/L) | CHOL  (g/L) |
| --- | --- | --- | --- | --- | --- |
| 1 | Male | 5 | 4.2 | 280 | 2.57 |
| 2 | Male | 7 | 4.8 | 350 | 1.88 |
| 3 | Male | 6 | 4.3 | 370 | 2.15 |
| 4 | Male | 5 | 4.9 | 270 | 2.62 |
| 5 | Male | 8 | 4.6 | 320 | 2.44 |
| 6 | Male | 5 | 5.0 | 420 | 2.01 |
| 7 | Male | 6 | 4.6 | 380 | 2.44 |
| 8 | Male | 5 | 4.9 | 440 | 3.36 |
| 9 | Male | 7 | 4.8 | 350 | 2.54 |
| 10 | Male | 8 | 5.1 | 520 | 2.33 |
| 11 | Male | 9 | 5.0 | 410 | 3.36 |
| 12 | Male | 6 | 4.7 | 370 | 2.17 |
| 13 | Male | 7 | 5.2 | 550 | 2.53 |
| 14 | Male | 5 | 5.1 | 460 | 2.55 |
| 15 | Male | 6 | 4.7 | 380 | 3.28 |
| 16 | Male | 7 | 5.3 | 410 | 2.18 |
