# Supplementary material for: Signature MicroRNA expression profile is associated with lipid metabolism in African green monkey
Source: Lipids Health Dis. 2019 Feb 28;18:55. doi: 10.1186/s12944-019-0999-2 (PMC6396449; doi:10.1186/s12944-019-0999-2)
Supplement: Supplementary file 2 — Table S2. Sequences of the primers used in the SYBR-green-based quantitative RT-PCR validation. (DOC 48 kb) [file 12944_2019_999_MOESM2_ESM.doc]

**Additional file 2: Table S2. Sequences of the primers used in the SYBR-green-based quantitative RT-PCR validation.**

| Primer name | Primer sequence (5′-->3′) |
| --- | --- |
| U6 RT | 5′CGCTTCACGAATTTGCGTGTCAT3′ |
| U6 fw | 5′GCTTCGGCAGCACATATACTAAAAT3′ |
| U6 rv | 5′CGCTTCACGAATTTGCGTGTCAT3′ |
| hsa-miR-9-5p RT | 5′GTCGTATCCAGTGCGTGTCGTGGAGTCGGCAATTGCACTGGATAC GACTCATAC3′ |
| hsa-miR-9-5p fw | 5′TCTTTGGTTATCTAGCT3′ |
| hsa-miR-9-5p rv | 5′GTGCGTGTCGTGGAGTCG3′ |
| hsa-miR-10b-5p RT | 5′GTCGTATCCAGTGCGTGTCGTGGAGTCGGCAATTGCACTGGATAC GACCACAAA3′ |
| hsa-miR-10b-5p fw | 5′TACCCTGTAGAACCGAA3′ |
| hsa-miR-10b-5p rv | 5′GTGCGTGTCGTGGAGTCG3′ |
| hsa-miR-122-5p RT | 5′GTCGTATCCAGTGCGTGTCGTGGAGTCGGCAATTGCACTGGATAC GACCAAACA3′ |
| hsa-miR-122-5p fw | 5′TGGAGTGTGACAATGG3′ |
| hsa-miR-122-5p rv | 5′GTGCGTGTCGTGGAGTCG3′ |
| hsa-miR-130b-5p RT | 5′GTCGTATCCAGTGCGTGTCGTGGAGTCGGCAATTGCACTGGATAC GACGTAGTG3′ |
| hsa-miR-130b-5p fw | 5′CACTCTTTCCCTGTTG3′ |
| hsa-miR-130b-5p rv | 5′GTGCGTGTCGTGGAGTCG3′ |
| hsa-miR-148a-3p RT | 5′GTCGTATCCAGTGCGTGTCGTGGAGTCGGCAATTGCACTGGATAC GACACAAAG3′ |
| hsa-miR-148a-3p fw | 5′TCAGTGCACTACAGAA3′ |
| hsa-miR-148a-3p rv | 5′GTGCGTGTCGTGGAGTCG3′ |
| hsa-miR-181a-5p RT | 5′GTCGTATCCAGTGCGTGTCGTGGAGTCGGCAATTGCACTGGATAC GACACTCAC3′ |
| hsa-miR-181a-5p fw | 5′CAACATTCAACGCTGTCG3′ |
| hsa-miR-181a-5p rv | 5′GTGCGTGTCGTGGAGTCG3′ |
| hsa-miR-185-5p RT | 5′GTCGTATCCAGTGCGTGTCGTGGAGTCGGCAATTGCACTGGATAC GACTCAGGA3′ |
| hsa-miR-185-5p fw | 5′TGGAGAGAAAGGCAGT3′ |
| hsa-miR-185-5p rv | 5′GTGCGTGTCGTGGAGTCG3′ |
| hsa-miR-370-3p RT | 5′GTCGTATCCAGTGCGTGTCGTGGAGTCGGCAATTGCACTGGATAC GACACCAGG3′ |
| hsa-miR-370-3p fw | 5′GCCTGCTGGGGTGGAA3′ |
| hsa-miR-370-3p rv | 5′GTGCGTGTCGTGGAGTCG3′ |
| hsa-miR-144-3p RT | 5′GTCGTATCCAGTGCGTGTCGTGGAGTCGGCAATTGCACTGGATAC GACAGTACA3′ |
| hsa-miR-144-3p fw | 5′TACAGTATAGATGA3′ |
| hsa-miR-144-3p rv | 5′GTGCGTGTCGTGGAGTCG3′ |
