# Supplementary material for: Signature MicroRNA expression profile is associated with lipid metabolism in African green monkey
Source: Lipids Health Dis. 2019 Feb 28;18:55. doi: 10.1186/s12944-019-0999-2 (PMC6396449; doi:10.1186/s12944-019-0999-2)
Supplement: Supplementary file 3 — Table S3. Primer sequences for miRNA-targeted genes. (DOC 40 kb) [file 12944_2019_999_MOESM3_ESM.doc]

**Additional file 3: Table S3 . Primer sequences for miRNA-targeted genes.**

| Primer name | Primer sequence (5′-->3′) |
| --- | --- |
| AGM ACAT1 fw | 5′TTGGGGAGGACGCCGAGGTCTT3′ |
| AGM ACAT1 rv | 5′TTTCCTGCACCAGCCTCCGGAG3′ |
| AGM ABCA1 fw | 5′AGCTGAGGTTGCTGCTGTGG3′ |
| AGM ABCA1 rv | 5′GCCGAACAGAGATCAGGATC3′ |
| AGM ABCG1 fw | 5′CATGTCACATGTGTTAGTGCA3′ |
| AGM ABCG1 rv | 5′GCTTGAGGACTAAAGAAAAG3′ |
| AGM AGPAT1 fw | 5′GTGAGATGGAGTCCTGGAGA3′ |
| AGM AGPAT1 rv | 5′GTAGCAGCATCCATGTCCCT3′ |
| AGM PPARg fw | 5′GTGAATTACAGCAAACCCCT3′ |
| AGM PPARg rv | 5′CTCTGTGTCAACCATGGTAATTTCT3′ |
| AGM SIK1 fw | 5′TGGTTATCATGTCGGAGTTC3′ |
| AGM SIK1 rv | 5′CAAGTTGCCTTTACCCAGGGT3′ |
| AGM CPT1A fw | 5′CATGGCAGAAGCTCACCAAGC3′ |
| AGM CPT1A rv | 5′TTCCAGGAATGAAGTCCAGA3′ |
| AGM IDH1 fw | 5′GTCCAAAAAAATCAGTGGCGG3′ |
| AGM IDH1 rv | 5′GCATCACGATTCTCTACGCC3′ |
| AGM FBXW7 fw | 5′GTGGAGTTACTGGGTCAGAG3′ |
| AGM FBXW7 rv | 5′GCAGTTCCTGATTCATTTCC3′ |
| AGM FASN fw | 5′CATGGAGGAGGTGGTGATTGC3′ |
| AGM FASN rv | 5′TCCGTAACCATGTCCACGCC′ |
